# Dancing Combines the Essence for Successful Aging

**DOI:** 10.3389/fnins.2012.00155

**Published:** 2012-10-11

**Authors:** C.-J. Olsson

**Affiliations:** ^1^Ageing and Living Conditions Programme, Centre for Population Studies, Umeå UniversityUmeå, Sweden; ^2^Umeå Centre for Functional Brain Imaging, Umeå UniversityUmeå, Sweden

In the scientific community much room has been given to the relationships between aging, cognition, and the brain. Unfortunately, there is strong evidence that aging is associated with impaired cognitive function (Rönnlund et al., 2005) as well as negative changes in brain structure (Salami et al., 2012) and in brain function (Nyberg et al., 2010). Consequently, with a worldwide population that is rapidly getting older the search for factors that facilitates successful aging, which in the present paper is referred to as maintained cognitive and brain functions, is a major and important challenge (Nyberg et al., 2012). In fact, there are suggestions regarding what can be done in order to maintain, or even improve, our memory functions over the lifespan. One line of research has focused on memory training. It has been shown that elderly engaged in a 5 week working memory training program improved their working memory function (Dahlin et al., 2008). However, it was also shown that the effect was limited to only the trained task for the elderly, and that only young participants showed evidence for transfer, i.e., an improvement in an untrained task, importantly, however, only to a task within the same cognitive domain. A second line of research has focused on the importance of a social network providing emotional and intellectual simulation in order to maintain cognitive and brain functions at older age (Fratiglioni et al., 2000). Moreover, this line of research has even suggested that absence of close social ties may be a risk factor for dementia. A third line of research has focused on how a physically active lifestyle may protect against age related cognitive decline. In several studies it has been shown how elderly that frequently engage in physical activity also have preserved brain structure (Erickson et al., 2009) as well as cognitive functions (Hillman et al., 2008), compared with elderly that do not take part in physical activities, especially cardiovascular activities. Moreover, it has also been shown that sedentary elderly individuals that starts to engage in moderate to low physical cardiovascular exercise actually improve their cognitive performance (Hillman et al., 2008), they increase their brain activity, measured by functional magnetic resonance imaging (Colcombe et al., 2004), and they even show a preserved and increased (!) volume of a brain structure that is important for memory, the hippocampus (Erickson et al., 2011). Thus, these separate lines of research suggests that factors such as keeping your memory system active, socializing, and being physically active may actually help to reduce the decline of cognitive functions associated with increased age.

Kattenstroth et al. (2010) hypothesized that *dance* would combine these different factors and provide superior sensory, motor, and cognitive performance in elderly that had been dancing for quite some time, compared with inactive elderly participants. To test the hypothesize they examined a group of elderly that could be categorized as amateur dancers with a longtime, over 16 years on average, history of dancing. They compared these individuals to a group of non-dancer controls, with no record of neither dancing nor sporting activities. There was no difference between the two groups in terms of education, gender or age. The authors measured a multitude of functions such as cognitive performance, reaction time performance, posture and balance, motor performance, and tactile performance investigating the hypothesis that long-year dancing activity promotes general advantages and preserves a multitude of body and mind functions. The results showed that in each of the different measured categories the dancing group outperformed the controls. The results from this study imply that the general decline in motor and cognitive performances often associated with increased age may be reduced if one engages in dancing during the life span. The effects were also reflected in every day competence such as independence, general health status, and life contentment. Interestingly, examining individual data it was clear that the effects were not a result of single individuals showing a superior behavior. Rather, individual data revealed that there were no individuals with a lifelong history of dancing that showed a poor performance on the different measures, as was observed from the participants without a dancing history.

An important feature of the Kattenstroth et al. (2010) study was that they were able to show the effects on multitude functions including cognition, posture, balance, as well as sensorimotor functions. Many studies have only measured functions within one category, for example cognition, and thus, have only been able to examine and make conclusions regarding isolated variables. In comparison, Kattenstroth and colleagues were able to show how dancing appears to have global effects on the body and on the mind.

Although the study by Kattenstroth et al. (2010) did not compare the results from their group of dancers with groups of elderly that have been engaging in other types of physical activities, it is tempting to suggest that dancing actually combines the essence for successful aging, at least in terms of maintained cognitive and brain functions. This can be hypothesized based on the fact that dance includes the three separate lines of research that has been suggested to promote brain functions including cardiovascular physical activity, cognitive training (as in learning new dance routines with different coordination), as well as social and emotional interactions. Thus, in future research it would be of interest to use dance as the intervention and to examine if similar beneficial effects could be reached in groups of elderly without prior history of dancing. Until then, the study by Kattenstroth and colleagues definitely proposes that one should gladly accept invitations for some turns and twists on a dance floor.
